# Enantiospecific ketoprofen concentrations in plasma after oral and intramuscular administration in growing pigs

**DOI:** 10.1186/1751-0147-54-55

**Published:** 2012-09-21

**Authors:** Katja Mustonen, Anneli Niemi, Marja Raekallio, Mari Heinonen, Olli AT Peltoniemi, Mari Palviainen, Mia Siven, Marikki Peltoniemi, Outi Vainio

**Affiliations:** 1 Department of Equine and Small Animal Medicine, Faculty of Veterinary Medicine, University of Helsinki, Helsinki, Finland; 2 Finnish Food Safety Authority Evira, Chemistry and Toxicology Unit, Helsinki, Finland; 3 Department of Production Animal Medicine, Faculty of Veterinary Medicine, University of Helsinki, Helsinki, Finland; 4 Division of Pharmaceutical Technology, Industrial Pharmacy, Faculty of Pharmacy, University of Helsinki, Helsinki, Finland; 5 Division of Bio pharmaceutics and Pharmacokinetics, Faculty of Pharmacy, University of Helsinki, Helsinki, Finland

**Keywords:** Non-steroidal anti-inflammatory drug, Swine, Enantiomer, Chirality, Pharmacokinetics

## Abstract

**Background:**

Ketoprofen is a non-steroidal anti-inflammatory drug which has been widely used for domestic animals. Orally administered racemic ketoprofen has been reported to be absorbed well in pigs, and bioavailability was almost complete. The objectives of this study were to analyze R- and S-ketoprofen concentrations in plasma after oral (PO) and intra muscular (IM) routes of administration, and to assess the relative bioavailability of racemic ketoprofen for both enantiomers between those routes of administration in growing pigs.

**Methods:**

Eleven pigs received racemic ketoprofen at dose rates of 4 mg/kg PO and 3 mg/kg IM in a randomized, crossover design with a 6-day washout period. Enantiomers were separated on a chiral column and their concentrations were determined by liquid chromatography-tandem mass spectrometry. Pharmacokinetic parameters were calculated and relative bioavailability (F_rel_) was determined for S and R –ketoprofen.

**Results:**

S-ketoprofen was the predominant enantiomer in pig plasma after administration of the racemic mixture via both routes. The mean (± SD) maximum S-ketoprofen concentration in plasma (7.42 mg/L ± 2.35 in PO and 7.32 mg/L ± 0.75 in IM) was more than twice as high as that of R-ketoprofen (2.55 mg/L ± 0.99 in PO and 3.23 mg/L ± 0.70 in IM), and the terminal half-life was three times longer for S-ketoprofen (3.40 h ± 0.91 in PO and 2.89 h ± 0.85 in IM) than R-ketoprofen (1.1 h ± 0.90 in PO and 0.75 h ± 0.48 in IM). The mean (± SD) relative bioavailability (PO compared to IM) was 83 ± 20% and 63 ± 23% for S-ketoprofen and R-ketoprofen, respectively.

**Conclusions:**

Although some minor differences were detected in the ketoprofen enantiomer concentrations in plasma after PO and IM administration, they are probably not relevant in clinical use. Thus, the pharmacological effects of racemic ketoprofen should be comparable after intramuscular and oral routes of administration in growing pigs.

## Background

Ketoprofen is a non-steroidal anti-inflammatory drug belonging to the 2-arylpropionic acid group. It has been widely used for domestic animals because of its anti-inflammatory, antipyretic and analgesic actions. In the European Union, the need of setting maximum residue limit (MRL) for ketoprofen for bovine, porcine and equine animals has been assessed and it has been concluded that ketoprofen can be included in the Annex II of the MRL regulation i.e. ketoprofen is not a subject to maximum residue limits [1]. The recommended dosage in intramuscular (IM) use in pigs is 3 mg/kg [2]. The oral solution has marketing authorization in several EU countries [3] for the treatment of fever and dyspnea associated with respiratory disease in fattening pigs at a dose rate of 1.5–3 mg/kg [4]. Orally administered racemic ketoprofen was reported to be absorbed well in pigs, and bioavailability was almost complete [5]. It also alleviated the signs of non-infectious lameness in sows [6].

Ketoprofen is a chiral compound existing in two enantiomeric forms, S (+) and R (−) ketoprofen. Each enantiomer has different pharmacodynamic and pharmacokinetic properties [7,8]. The S-enantiomer is a more potent cyclooxygenase inhibitor than the R-enantiomer [9], while the R-enantiomer has a potent analgesic effect that does not involve cyclooxygenase inhibition [10,11]. The commercial products in veterinary medicine are 50:50 racemic mixtures of both enantiomers.

The enantiospecific pharmacokinetics of ketoprofen have been studied in several food-producing animal species. In most species, including horses [12] and pigs [13,14], S-ketoprofen is the predominant enantiomer in the plasma after administration of the racemic drug, whilst R-ketoprofen is predominant in sheep [15]. The plasma concentrations of both enantiomers are similar in goats [16] and calves [17].

In humans, rats and mice [18-21], the absorption of ketoprofen is not stereoselective, but low enantioselectivity has been found in first-pass metabolism in humans [22]. However, in rats and cats, enantioselective pharmacokinetics were influenced by the oral route of administration compared to intravenous administration [21,23], whereas in elephants there were no significant differences [24]. In our previous study a second peak in total ketoprofen concentrations in plasma was observed in pigs [5], which may indicate enterohepatic circulation of the drug. Stereoselective enterohepatic circulation of ketoprofen has been reported to exist in rats [25].

In addition to enantiospecific disparities in the distribution and elimination processes, pharmacokinetic differences between the enantiomers are caused by chiral inversion. Ketoprofen undergoes unidirectional chiral inversion from the R- to the S- enantiomer. The extent of inversion varies considerably between species. In pigs the inversion rate is reported to be 70% [26]. The extent of inversion is not affected by the dose rate [22,27]. Administration of racemic ketoprofen instead of a pure enantiomer has an influence on the enantiomer concentration ratio in plasma [15,17,18,28].

In clinical practice, intramuscular injections and per oral administration are the most common routes to medicate pigs. The objectives of this study were to assess the relative bioavailability of racemic ketoprofen for both enantiomers between IM and PO administration in pigs and to analyze the R- and S-ketoprofen concentrations in plasma after both routes of administration. Our secondary aim was to confirm the existence of double peaks in enantiospesific ketoprofen concentrations in plasma.

## Materials and methods

### Animals

Eleven crossbred pigs (5 females and 6 barrows) from a specific pathogen-free production herd were used. They were 9–13 weeks old at the beginning of the trial and their mean body weights (± SD) were 36.5 ± 2.8 kg at the beginning and 42.6 ± 3.2 kg at the end of the trial. In vivo procedures were undertaken at the Department of Production Animal Medicine, University of Helsinki. The study was approved by the Finnish National Animal Experiment Board. The pigs were housed individually in pens. Standard commercial pellet feed for growers containing no antimicrobials was offered twice daily and water was supplied ad libitum. The health status of the animals was assessed on the basis of clinical examination, hematology and blood chemistry prior to the study procedures and before the second drug administration.

A vinyl tube (inner diameter, 1.0 mm; outer diameter 1.5 mm, Biocorp Australia Pty Ltd, Australia) for blood sampling was inserted nonsurgically into the ear vein or vena cephalica one day before the first sampling [5]. During the wash-out period, the vinyl tubes were flushed with heparinized saline twice a day to prevent blood clots forming inside them.

### Study design and procedures

The study utilized a balanced crossover design. All pigs received two treatments in a random order with a six-day wash-out period between the treatments. Ketoprofen was administered orally (Ketovet, Galena, Kuopio, Finland) at a dose of 4 mg/kg or intramuscularly (Romefen, Merial, Lyon, France) at dose of 3 mg/kg. Oral powder was mixed with 40 mL of tap water and administered via a stomach tube, which was flushed with 50 mL of water before removal. The injection site for intramuscular administration was the muscle on the right side of the neck, approximately 5 cm caudally from the base of the ear. Injections were performed using single-use 18 G needles and 2 mL syringes. The pigs were fasted overnight before treatments and fed 2 hours post-dosing. Water was offered ad libitum.

Blood samples were collected before treatment and at 0.5, 0.75, 1, 1.25, 1.5, 1.75, 2, 4, 6, 8, 10, 12, and 24 hours post-treatment. To prevent contamination of the sample with heparinized saline, the first 2 mL drawn up was discarded and the sample was taken with an unused 10 mL syringe and placed into 10 mL heparinized tubes. Plasma was separated by centrifugation (2500 rpm, 10 min) within 1 hour and stored at −20°C until analyzed.

### Analysis

The analysis method used is an unpublished in-house method based on the modification of the Oasis MAX application [29]. Enantiomers were separated on a chiral column and detected by liquid chromatography-tandem mass spectrometry (LC-MS/MS) analysis. Organic solvents were of high-performance liquid chromatography grade and other chemicals of analytical purity. Matrix-matched calibration curves were based on the 50:50 racemic mixture. Racemic ketoprofen (U.S. Pharmacopeia, Rockville, USA) was used for standard curves and spiked samples, and the corresponding deuterium-labeled ketoprofen-d4 was used as an internal standard (QMX Laboratories, Thaxted, Essex, UK). Optically active S-ketoprofen (Aldrich, St. Louis, USA) was used to identify the elution order of ketoprofen enantiomers. Appropriate standard solutions were prepared by dilution with methanol.

For ketoprofen analysis a portion of each sample (0.5 mL) was taken. An internal standard working solution (50 μl, 1 μg/mL) was added to the sample, which was then diluted with ammonia (4%, 0.5 mL), subsequently followed by solid phase extraction (Oasis MAX, 1cc, 30 mg, Waters, Milford, USA). Before sample loading, the cartridge was conditioned with methanol (1 mL) and water (1 mL). The cartridge was washed with ammonia (5%) in water (1 mL), methanol (1 mL) and formic acid (2%) in methanol/water (45/55, v/v, 1 mL). The analytes were eluted with formic acid (2%) in methanol/water (90/10, v/v, 1 mL). The solvent was evaporated to dryness under a stream of nitrogen at 45°C and redissolved in a mobile phase (250 μL). An aliquot of each sample (10 μL) was injected into the LC-MS/MS.

Ketoprofen enantiomers were separated on a chiral column (Chirobiotic R, 15 cm x 2.1 mm, 5 μm, Supelco, Bellefonte, USA) protected by cartridges (4x 2.0 mm, Security Guard C 18, Phenomenex, Cheshire, UK) using ammonium acetate buffer (20 mM, pH 5.6 adjusted with formic acid) in 30% methanol. The flow rate was 0.2 mL/min and the column temperature was set at 18°C.

The LC-MS/MS instrumentation consisted of a separation module (Waters Alliance 2695 Separation Module, Waters, Milford, USA) and a triple quadrupole tandem mass spectrometer (MicroMass Quattro Micro, MicroMass Ltd., Manchester, UK) operated in the negative ion mode. The multiple reaction monitoring mode was used for acquiring data. The deprotonated molecular ion [M-H]^-^ was m/z 253.2, and the product ions were m/z 209.3 and m/z 197.1 when the collision energy was 9.0 eV. The transition m/z 257.3 to m/z 213.3 was monitored for internal standard. Peak integration and calibration were performed using commercial software (MassLynx 4.0, Waters, Milford, USA).

The matrix-matched calibration curve was found to be linear over the selected concentration range of 5–7000 ng/mL for each enantiomer by a weighted least-squares linear regression model. Each point of the calibration curves was tested on its acceptable linearity with the method of van Trijp and Roos [30]. The correlation coefficients for the calibration regression curves were 0.996 or greater.

Recovery and precision (repeatability and within-laboratory reproducibility) were measured by spiking six blank plasma samples at levels of 10, 30, and 100 ng/mL of ketoprofen as the racemate on three different days (N = 18 for all concentrations). Recoveries were determined by comparing the measured concentrations of spiked samples with corresponding nominal concentrations. Recoveries varied between 94–109% for S-ketoprofen and 91–96% for R-ketoprofen. The intra- and inter-day precisions (%CV) were less than 12.9% and 11.2% for both analytes. The specificity was tested by analyzing blank plasma samples for the presence of interfering compounds. There was no significant interference from endogenous substances in blank plasma samples at the retention times of the analytes and internal standard, demonstrating the specificity of the method. Blank plasma samples spiked with different levels of ketoprofen as the racemate (40, 100 and 500 ng/mL) were used as quality control samples in each sample set.

The ion suppression was studied by comparing the responses to matrix calibration standards with those standards having corresponding concentrations of the ketoprofen racemate but without matrix. According to our results, the difference in the responses observed with and without the matrix was insignificant. The limit of quantification (LOQ) for both enantiomers was defined to be 5 ng/mL by estimating from spiked blank plasma samples at the lowest validation level at the signal-to-noise ratio of 10.

### Pharmacokinetic parameters

Pharmacokinetic parameters were calculated (Kinetica software, Thermo Electron Corp, Waltham, USA). The non-compartment analysis was applied for R- and S- ketoprofen plasma disposition curves. The area under the plasma concentration-time curve (AUC) was calculated by use of the trapezoidal method. In each case, AUC_0–24_ was >80% of the calculated AUC_0–∞_. Values for the maximum plasma concentration (C_max_) and time to peak plasma concentration (T_max_) were directly determined from individual time vs. plasma concentration curves. The terminal half-life (T_1/2_) was calculated as 0.693/β, in which β is the terminal rate constant. The mean residence time (MRT) was calculated as the area under the first-moment curve from time 0 to infinity divided by AUC_0–∞_. The relative bioavailability (F_rel_) was determined for S and R –Ketoprofen by calculating (AUC_po_/ AUC_im_) (D_im_/D_po_)*100%. IM route was used as a reference.

### Statistical analysis

Pharmacokinetic parameters obtained for S- and R- ketoprofen within administration groups were compared by use of the paired Wilcoxon *t-*test (AUC, C_max_, T_1/2_, MRT) or Wilcoxon matched-pairs rank test (T_max_) (Kinetica software, Thermo Electron Corp, Waltham, USA). As ketoprofen pharmacokinetics is linear [5], the dose was normalized (AUC_norm_ = (D_im_/D_po_) x AUC_po_) when AUCs were statistically compared between administration groups. Differences were considered significant at *P* < 0.05.

## Results

**Figure 1:**
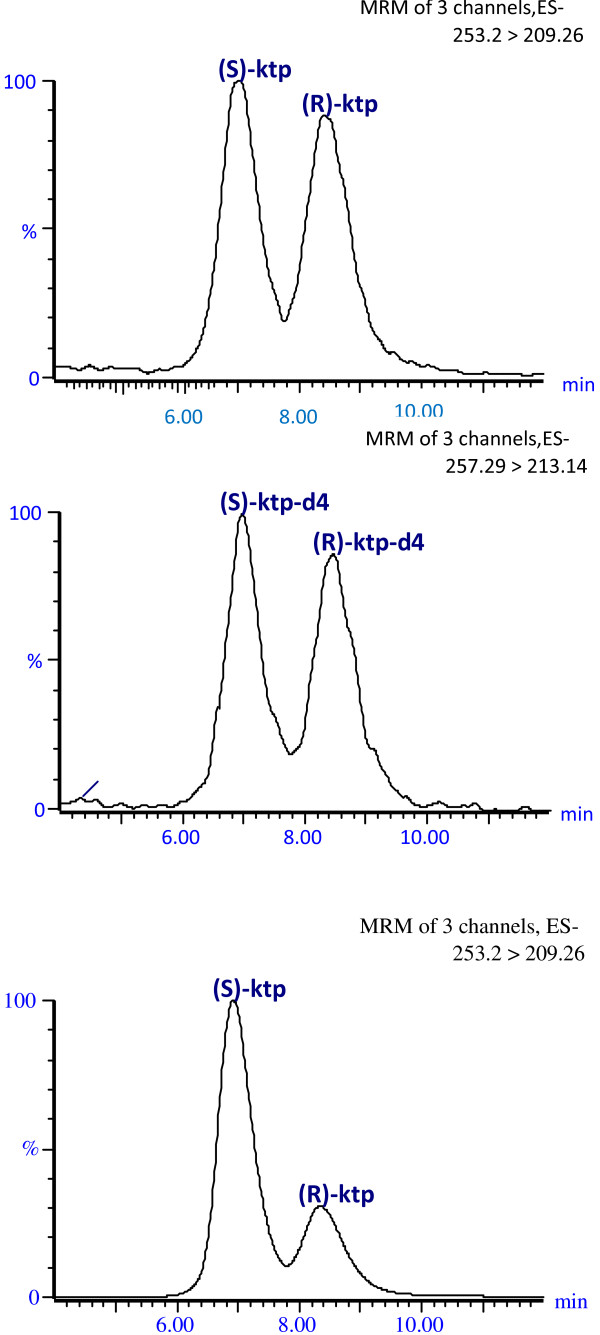
LC-MS/MS chromatograms of (A) porcine plasma spiked with racemic ketoprofen (100 ng/mL for each enantiomer) and (B) the corresponding internal standard (50 ng/mL for each enantiomer), and (C) a plasma sample obtained from a pig at 45 minutes after oral administration of racemic ketoprofen (4 mg/kg).

Representative ion chromatograms of porcine plasma spiked with 200 ng/mL for both enantiomers, including the deuterated internal standard and a plasma sample collected from a pig at 45 minutes after oral administration of racemic ketoprofen (4 mg/kg), are presented in Figure 1. The peaks corresponding to the S- and R-ketoprofen retention time were 7.0 min and 8.4 min, respectively.

**Figure 2:**
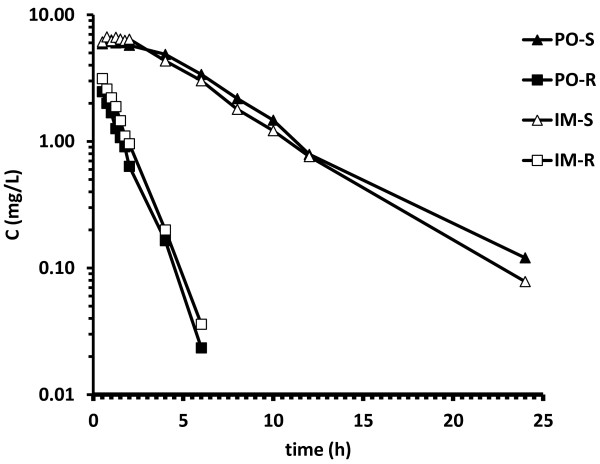
**Mean ± SEM plasma concentrations of S- and R-enantiomers in 11 pigs after administration of single doses of racemic ketoprofen, 3 mg/kg IM and 4 mg/kg PO in cross-over design.** Ketoprofen was administered at time 0.

**Figure 3:**
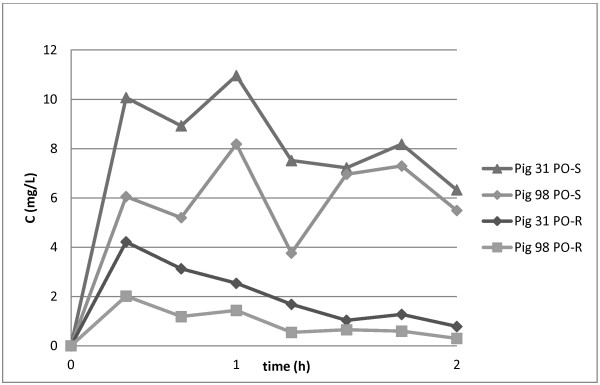
Individual plasma S- and R-ketoprofen concentration profiles of two pigs the first two hours after PO administration of racemic ketoprofen at dose rate of 4 mg/kg.

The mean S- and R-ketoprofen concentration profiles in plasma following oral and intramuscular administration are illustrated in Figure 2. The S-ketoprofen concentration in plasma was above the LOQ for all sampling points after drug administration, except in one pig after the PO route and three pigs after the IM route at 24 hours post-administration. In most pigs (seven animals after PO administration and nine after IM administration), R-ketoprofen was above the LOQ for four hours or less. The remaining animals had quantifiable values for up to 24 hours. The second peak was evident in S-ketoprofen concentrations in plasma in most pigs and it was more obvious after PO administration than after IM administration. It was not detected in R-ketoprofen (Figure 3).

Pharmacokinetic parameters for plasma S- and R-ketoprofen are presented in Table 1. No carry-over was detected. The differences between the corresponding enantiomers within each route of administration were significant with respect to all parameters analyzed. Between the routes of administration, the differences with respect to C_max_, T_max_ ,T_1/2_ and MRT were not significant. F_rel_ (%) was 83% ± 20% and 63% ± 23% (*P* = 0.01) for S-ketoprofen and R-ketoprofen, respectively. When comparing the administration routes, the differences between AUCs of both enantiomers as well as the AUC_S_/AUC_R_ ratio were significant.

**Table 1:** Mean values (± SD) of pharmacokinetic parameters for ketoprofen after PO or IM administration of a single dose in a cross-over design to 11 crossbred pigs

		**Route of administration**	
**Parameter**		**PO 4 mg/kg**	**IM 3 mg/kg**
C_max_ (mg/L)	S-Ketoprofen	7.42 ± 2.35^*^	7.32 ± 0.75^*^
	R-Ketoprofen	2.55 ± 0.99	3.23 ± 0.70
T_max_ (h)	S-Ketoprofen	1.91 ± 1.65^*^	1.27 ± 0.45^*^
	R-Ketoprofen	0.59 ± 0.23	0.65 ± 0.30
AUC_0-∞_ ([mg/L] h)	S-Ketoprofen	47.04 ± 13.41^*^	44.09 ± 12.87^*^
	R-Ketoprofen	3.83 ± 1.23	5.16 ± 2.48
AUC_0-24_ ([mg/L] h)	S-Ketoprofen	46.26 ± 12.77*	43.46 ± 12.88*
	R-Ketoprofen	3.54 ± 1.17	4.53 ± 2.19
AUC_24-∞_ (%)	S-Ketoprofen	1.56 ± 1.63	1.55 ± 1.77
	R-Ketoprofen	7.13 ± 8.83	12.06 ± 10.12
T_1/2_ (h)	S-Ketoprofen	3.40 ± 0.91^*^	2.89 ± 0.85^*^
	R-Ketoprofen	1.1 ± 0.90	0.75 ± 0.48
MRT (h)	S-Ketoprofen	5.55 ± 1.45^*^	4.91 ± 1.21^*^
	R-Ketoprofen	1.73 ± 0.92	1.49 ± 0.67
AUC_S_/AUC_R_ ratio		12.8 ± 3.17^**^	9.2 ± 2.39

* Denotes a significant difference (P < 0.05) between S-ketoprofen and R-ketoprofen when comparing within treatments.

** Denotes a significant difference (P < 0.05) between administration routes (PO versus IM).

## Discussion

S-ketoprofen was the predominant enantiomer in pig plasma after administration of the 50:50 racemic drug via both routes. The pharmacokinetic parameters of both enantiomers after IM administration were similar to those reported by Fosse [31] in piglets, despite the pigs in our study being older than those used in their study. T_max_ and AUC_S_/AUC_R_ ratio after oral administration were higher in our study than in the study reported by Neirinckx [14]. The ketoprofen product used in that study was an oral solution, which could have been absorbed faster than the oral powder we used.

The absolute bioavailability of both ketoprofen enantiomers after IM administration is suggested to be almost complete [13,31]. After PO administration the bioavailability is reported to be approximately 85 % for both enantiomers [14]. The higher relative bioavailability for S than R ketoprofen in our study suggests that some stereoselective absorption and/or first pass metabolism may have occurred. The physiochemical properties of the two enantiomers of ketoprofen are identical and absorption has been regarded to be mainly a passive process. The absorption of ketoprofen is therefore not considered to be stereoselective [18-21,32]. However, there is also some evidence suggesting that ketoprofen may have an active transport pathway across the intestinal wall [33]. In rats, 84% of the administered dose of R-ketoprofen was inverted to S-ketoprofen in the gastrointestinal tract [21], while an absence of pre-systemic inversion was reported in pigs [26]. Presystemic inversion in the gastrointestinal tract has also been reported in rats after ibuprofen and fenoprofen administration, and was dependent on the absorption rate [34,35]. In the present study, the difference in relative bioavailability between enantiomers was smaller, although significant, than that reported in rats after PO and IP administration [21]. The inversion rate of R-ketoprofen to S-ketoprofen is equally high (approximately 70%) in rats and pigs [18,26]. The possible faster absorption rate of the oral solution used by Neirinckx [26] may have been partially responsible for the absence of pre-systemic inversion found in their study, whereas in our study the slower absorption rate from the gastro-intestinal tract might have contributed the pre-systemic inversion. The racemic ketoprofen used in our study was an oral powder, which was insoluble in water.

The second peak in S-ketoprofen concentration in plasma after PO and IM administration maybe due enterohepatic recycling. There were individual variation in the sharpness of the second peak and the peak was more evident after PO administration of ketoprofen than after IM administration. In most of the pigs, it was clearly evident within two hours after per oral ketoprofen administration. That confirms our previous findings with total ketoprofen plasma concentrations [5]. The stereoselective enterohepatic circulation of ketoprofen exists at least in rats [25]. Yasui [25] suggested that glucoronide of S-ketoprofen is hydrolyzed slower than the glucuronide of R-ketoprofen in the intestine. That will lead to longer mean transit time of S-ketoprofen from the bile duct via the intestinal tract and into the systemic circulation and therefore stereoselective enterohepatic recycling may occur. However, the high degree of chiral inversion from S- to R-ketoprofen could also explain some fluctuation in plasma S-ketoprofen concentration.

Since the difference in AUC values between enantiomers was significant for both administration routes, one of the most probable reasons for the lower AUC of R-ketoprofen was higher clearance compared to S-ketoprofen. The enantioselectivity differences in clearance may be attributable to differences in distribution, chiral inversion, hepatic metabolism, renal excretion, or to a combination of these factors. The volume of distribution is low for ketoprofen in pigs, probably due to high protein binding [13]. The degree of stereo selectivity in binding to plasma or tissue proteins, which is species dependent, may result in a significant effect on the amount of drug in the plasma [36]. Enantioselectivity in the binding of ketoprofen to plasma proteins has been reported in humans and camels [37,38], although contradictory results have also been reported in humans [39]. There have been no reports of possible enantioselectivity protein binding in pigs.

The terminal half-life and MRT of R-ketoprofen were approximately three times shorter and T_max_ a half of that for S-ketoprofen after both administration routes. In the present study, pure enantiomers were not administered and chiral inversion and the inversion rate could not be determined. The inversion rate from R-ketoprofen to S-ketoprofen has been previously reported to be 70% in pigs [26]. Ketoprofen is metabolized in the liver and converted into a carbonyl-reduced derivative, 2-(phenyl 3-alphahydroxybenzoyl) propionic acid in swine [1]. In rats, stereo selectivity has been reported for the biliary excretion process [40]. The significance of stereo selectivity in reductive metabolism is difficult to assess, as inversion occurs in most species, and is rapid for ketoprofen [41]. Glucuronidation appears to be an important metabolic pathway for ketoprofen in pigs [1]. Stereo selectivity of glucuronidation has been reported, but it is species and compound dependent [42]. The possible stereo selectivity of ketoprofen glucuronidation in pigs has not been studied, but it could to some extent explain the more rapid elimination of R-ketoprofen than S-ketoprofen.

Fosse [31] reported an IC_50_ for S-ketoprofen of 26.7 μg/mL and an IC_50_ for R-ketoprofen of 1.6 μg/mL from mechanical nociceptive threshold testing in the kaolin-induced inflammation model in neonatal pigs. There have been no other reports on either the total or the enantiospecific therapeutic target concentration of ketoprofen in plasma in pigs. A total ketoprofen plasma concentration of 0.4–6 μg/mL has been recommended as a target therapeutic concentration in humans [43]. A serum concentration of 0.2–0.4 μg/mL of S-ketoprofen is required for the maximum anti-inflammatory effect in adjuvant arthritis in rats [32], whereas at least 1 μg/mL of total ketoprofen is needed to alleviate pain in orthopedic human patients [44]. In the present study the S-ketoprofen concentrations in plasma in this study were above 0.8 μg/mL for at least 12 hours. The concentration of 1.6 μg/mL of R-ketoprofen was only achieved for two hours. Fosse [31] reported a biphasic analgesic effect in piglets; an initial comprehensive but short analgesia followed by a moderate but more sustained analgesia. The authors hypothesized that the former was caused by R-ketoprofen and the later by S-ketoprofen. Ketoprofen concentrations persist for longer in inflammatory exudates than in plasma [15,17,28,45,46], and the clinical effect may therefore last longer than estimated from concentrations in plasma.

In the European Union, the registered ketoprofen dose rate for pigs is 3 mg/kg body weight IM [2] or 1.5–3 mg/kg body weight PO [4]. In our previous study, increases in AUC and C_max_ were proportional in orally administered doses (3 mg/kg and 6 mg/kg) of racemic ketoprofen when the total plasma ketoprofen was measured [5]. However, equivalence was not detected between 3 mg/kg PO and IM. Accordingly, we estimated that the oral dose used in this study, 4 mg/kg, would produce a maximum concentration in plasma similar to the registered dose for intramuscular administration (3 mg/kg).

The relative bioavailability of S-ketoprofen after oral administration was significantly higher than for R-ketoprofen. Since S-ketoprofen is generally regarded as the eutomer regarding cyclooxygenase inhibition, and the terminal half-life of R-ketoprofen is short, in clinical use the administration routes at dose rates used in this study could be considered equally effective.

## Competing interests

The authors declare that they have no competing interests.

## Authors’ contributions

KMM, MRR, MLH, OATP and OMV designed the study protocol. KMM, MRR, MLH and OATP performed the clinical study procedures. MPa and AN prepared and analyzed the samples. MSS and MPe performed pharmacological and statistical analyses. KMM, AN and MRR drafted the manuscript, and all authors contributed to, and approved, the final manuscript.
